# External validation of BIA equations to estimate appendicular skeletal muscle mass in older adults: Importance of the bias analysis and derivation of correction factors to achieve agreement

**DOI:** 10.3389/fnut.2022.951346

**Published:** 2022-08-25

**Authors:** María Cáñez-Ríos, Julián Esparza-Romero, Rogelio González-Arellanes, Maribel Ramírez-Torres, Guadalupe Figueroa-Pesqueira, René Urquidez-Romero, Diana Beatriz Rangel-Peniche, Heliodoro Alemán-Mateo

**Affiliations:** ^1^Coordinación de Nutrición, Centro de Investigación en Alimentación y Desarrollo (CIAD), A.C., Hermosillo, Mexico; ^2^Departamento de Ciencias de la Salud, Instituto de Ciencias Biomédicas, Universidad Autónoma de Ciudad Juárez, Ciudad Juárez, Mexico; ^3^Coordinación de Nutrición, Universidad Estatal de Sonora, Hermosillo, Mexico; ^4^Facultad de Ciencias Naturales, Licenciatura y Maestría en Nutrición, Campus Juriquilla, Universidad Autónoma de Querétaro, Querétaro, Mexico

**Keywords:** appendicular skeletal muscle mass, non-Caucasian older adults, predictive equations, bioimpedance analysis, dual-energy X-ray absorptiometry, external validation

## Abstract

There are several equations based on bioelectrical impedance analysis (BIA) to estimate with high precision appendicular skeletal muscle mass (ASM). However, most of the external validation studies have reported that these equations are inaccurate or biased when applied to different populations. Furthermore, none of the published studies has derived correction factors (CFs) in samples of community-dwelling older adults, and none of the published studies have assessed the influence of the dual-energy X-ray absorptiometry (DXA) model on the validation process. This study assessed the agreement between six BIA equations and DXA to estimate ASM in non-Caucasian older adults considering the DXA model and proposed a CF for three of them. This analysis included 547 non-institutionalized subjects over 60 years old from the northwest of Mexico who were physically independent and without cognitive impairment: 192 subjects were measured using DXA Hologic, while 355 were measured by DXA Lunar. The agreement between each of the equations and DXA was tested considering the DXA model used as a reference method for the design of each equation, using the Bland and Altman procedure, a paired *t* test, and simple linear regression as objective tests. This process was supported by the differences reported in the literature and confirmed in a subsample of 70 subjects measured with both models. Only six published BIA equations were included. The results showed that four equations overestimated ASM_DXA_, and two underestimated it (*p* < 0.001, 95% CI for Kim's equation:−5.86-−5.45, Toselli's:−0.51-−0.15, Kyle's: 1.43–1.84, Rangel-Peniche's: 0.32–0.74, Sergi's: 0.83–1.23, and Yoshida's: 4.16–4.63 kg). However, Toselli's, Kyle's and Rangel-Peniche's equations were the only ones that complied with having a homogeneous bias. This finding allowed the derivation of CFs, which consisted of subtracting or adding the mean of the differences from the original equation. After estimating ASM applying the respective CF, the new ASM estimations showed no significant bias and its distribution remained homogeneously distributed. Therefore, agreement with DXA in the sample of non-Caucasian was achieved. Adding valid CFs to some BIA equations allowed to reduce the bias of some equations, making them valid to estimate the mean values of ASM at group level.

## Introduction

Skeletal muscle performs a broad range of mechanical, structural and metabolic functions (1). It increases from childhood and remains constant between 18 and 40 years. From the age of 45, the skeletal muscle mass is progressively lost (2). The loss of skeletal muscle is associated with malnutrition, sarcopenia, loss of functionality and other adverse effects in older adults (3–7). Therefore, it should be a priority to assess this body composition component in this age group. Currently, there are several precise and accurate methodologies to measure skeletal muscle such as magnetic resonance imaging (MRI), computed tomography, dual-energy X-ray absorptiometry (DXA), and deuterated creatine (D^3^-creatine) dilution. However, they are expensive and not feasible or available for regular epidemiological or clinical practice. A less expensive, non-invasive, and reliable alternative method is bioelectrical impedance analysis (BIA). From this methodology, variables such as resistance and reactance can be obtained. These variables, together with other anthropometric or sociodemographic variables, can be included in BIA predictive models or equations to estimate total or appendicular skeletal muscle mass (ASM) considering MRI or DXA as reference methods.

Various predictive models based on BIA have been developed worldwide (8–17). Some have been validated through internal (8, 10, 11, 15, 17) or external validation procedures (18–20) to estimate ASM in older adults. However, high values of the coefficient of determination (*R*^2^), low standard error of the estimate (SEE) of the predictive model, or the results of internal or external validation in a particular group, do not guarantee the validity of the predictive models to estimate ASM in other populations with specific or different characteristics (11, 12, 14, 16, 20–22). Very few studies have considered valid some BIA equations to estimate ASM within the same ethnic group (18), or with different health conditions (19, 23). In general, it is recognized that BIA equations that estimate ASM or any other body composition compartment are only precise, accurate and unbiased in populations with similar characteristics to the sample or ethnic group where it was generated (24, 25). These findings and others support that the published BIA equations should not be applied interchangeably. They also highlight the need for external validation in the population of interest. This validation procedure will determine whether or not the predictive models could be generalized (26).

Currently, it is noticeable that most studies aim to generate new precise models to estimate body composition components or compartments based on the assumption (8, 10) and their own results (9, 11, 12, 14, 16) that the existing models are not valid in certain populations. Meanwhile, in other studies, it is possible to notice the efforts to use the existing equations, and in this way, avoid the generation of new ones unjustifiably (18, 19, 23, 27, 28). However, based on the results of these studies, some of them have resulted inaccurate or have not achieved agreement. In this case, an effective strategy may be the analysis of the bias during the validation process. This analysis consists on evaluating the trend of bias: verifying that it remains constant regardless of the amount of ASM presented. This allows discarding the use of published predictive equations, provide the bases for the development of new age, gender, or ethnic specific BIA equations, or determine the possibility of generating CFs for existing equations. These CFs are derived from the mean differences between the equations' estimates and the measurements of the reference method. The condition to derive one, is that the bias must be distributed homogeneously throughout the average of both methods. If it meets this criterion, it will be possible to add or subtract the mean difference to the estimated value of the original equation. In this way, it is possible to achieve agreement. However, it is important to clarify that a simple correction factor might not eliminate the prediction error in individuals, since these data come from the values of *R*^2^ and the standard error in the estimation of the original equation.

In the case of Mexico, there is only one study (11) where two published BIA equations were applied to estimate ASM in healthy non-institutionalized older adults. The results of this external validation study showed that Kyle's and Sergi's equations were inaccurate in the validation sample. Likewise, the researchers generated, and internally validated a new specific BIA equation for older Mexican adults from the center of the country. In the aforementioned study, the bias of the Kyle and Sergi's equations was not explored, and it has not currently been explored whether the equation generated in older adults from the center of Mexico and other published BIA equations could be valid for older adults from the northwest of Mexico. This, taking into account that it was previously reported that older adults from the northwest of Mexico had less ASM compared to those from the center of the country (29). This may probably be due to differences in total and central fat. Women and men from northwestern Mexico were fatter than those from central Mexico. A positive association has been shown between fat mass and some markers of inflammation, such as C-reactive protein (CRP), and a negative association between CRP and ASM (30).

In general, there are no studies where the equations' bias has been critically analyzed, nor where correction factors have been proposed for the existing equations based on BIA to estimate ASM. This could stop the generation of models that may never be used. The external validation and bias analysis can provide alternatives and close the gap between equation development and implementation of equations, in this case, for estimation at the group level (26).

Moreover, the influence of the DXA model used as reference method, is a factor which has not been explored in external validation studies. Currently, the most widely used models are DXA Lunar and DXA Hologic, of which significant differences in body composition measurements have been reported between both models (31, 32). Considering this evidence, it is possible that the ASM estimated by an equation may not be entirely comparable or equivalent when compared to ASM measurements with a different DXA model than the one used for the generation of the equation. This could lead to bias in external validation studies. For all of the above, the objective of this study was to assess the agreement between six equations based on BIA and dual-energy X-ray absorptiometry to estimate ASM in non-Caucasian older adults, considering the DXA model. The bias was also analyzed in order to propose correction factors.

## Materials and methods

This is a secondary analysis generated from various studies with a cross-sectional design (33–35) and the baseline data of one randomized clinical trial (36) carried out in the Body Composition Laboratory of the Food and Development Research Center, (CIAD, A.C.). This analysis included a large sample of older men and women from Hermosillo, Sonora, México. The methodology has already been described previously in the mentioned studies, but a brief description is provided.

### Subjects

Independently of the cited studies, all participating subjects were adults over 60 years of age or older, who were invited to participate through flyers, telephone calls and home visits. The corresponding study protocol was explained to them, as well as the procedures to which they would undergo. All volunteers underwent body composition measurement by different methodologies including DXA and BIA. The subjects were categorized according to their body mass index (BMI, kg/m^2^) using to the WHO classification (37). Likewise, various questionnaires and scales were applied to determine the health status, including functionality and cognition. All the subjects were free of physical disability according to the Lawton and Brody scale (38) or the Barthel Index (39), and the majority were free of cognitive impairment according to the Pfeiffer Scale (40) or the Mini Mental State Examination (41). Also, information on demographic and socioeconomic conditions was collected. All these procedures were conducted at CIAD, A.C. From the cited studies, a primary database was built.

All the volunteers selected for this study, had to have a physical file, which had to contain complete information on age, sex, waist circumference, resistance and reactance variables, and DXA scans. They had to be free of diseases, conditions or medications that could affect body composition or hydration status. Regardless of their BMI, men and women older than 60 years were included. All those subjects who did not have complete data on the variables necessary for this external validation protocol, and those who had atypical data or outliers detected by the exploratory analysis were excluded. The identification of outlier variables was carried out through the visual identification of variables that were separated from the set of points of the scatter plot.

#### Anthropometry

Body weight was measured without shoes and minimum of clothes or disposable gown and recorded by the HV-200KGL scale (A&D Weighing, CA, US), which was previously calibrated with a known weight. Height was measured in the same condition, placing the subject's head according to the Frankfurt plane and using a digital stadiometer (SECA stadiometer 274, Hamburg, Germany). Afterwards, the BMI was calculated. Waist circumference was measured just above the superior border of the iliac crest. The measurement was made with the subject standing and using a fiberglass measuring tape (Lafayette Instruments Company Inc., Lafayette, IN, USA).

#### Appendicular skeletal muscle mass measurements

Body composition in some of the cited studies was assessed using DXA Lunar Radiation Corp; Madison, WI, USA or DXA Hologic Discovery WI QDR Series; Waltham, USA. It is important to point out that it has been reported that, quantitatively, these two models do not measure the exact same amount of body composition components such as ASM, or compartments such as fat mass. Regarding the appendicular lean mass (ALM), Shepherd et al. (31) showed significant differences in the measurement of ALM by both models (16.176 kg using Hologic Discovery vs. 15.715 kg with GE Lunar, *p* < 0.01). For this study, we analyzed the ASM measurements in a subsample of 70 older adults, who had been measured with both DXA models. DXA measurements were performed in the same day, following the same protocol for the whole scan and scan editing for ASM determination. A paired *t*-test was used to determine if the mean difference of the measurements between both methods was different from zero.

Protocol of DXA measurement, DXA scan edition for ASM and calibration were performed according to a published study (42). Participants were measured wearing a disposable gown and free of plastic or metal objects. The ASM determined by DXA (ASM_DXA_) was considered as reference. For those that did not fit in the DXA scan area, half-body scans were performed, and the remaining side was duplicated as described by Rothney et al. (43). In the case of two subjects who wore non-removable metal accessories, the opposite half of the body to where they had the accessory, was duplicated. In addition to ASM, fat mass measured by DXA was considered to estimate the fat mass index (kg of fat/height in m^2^).

#### Bioimpedance analysis

For the purposes of this secondary analysis, resistance (R) and reactance (Xc) were measured by a RJL Systems single frequency bioimpedance (50 kHz), Detroit, Mich, USA, which complied with a daily calibration protocol with a resistance of 500 ohms. BIA measurements were according to the methodology published previously (24, 44). Both the DXA and BIA measurements were performed after an 8 h fast, with an empty bladder and without having consumed food or liquid prior to the measurement.

### Selected BIA predictive models

English-language articles on topics of BIA equations or predictive models to estimate ASM published between 2000 and 2022 were identified in the PubMed database. The keywords for the search were “appendicular skeletal muscle mass,” “muscle mass,” “BIA equation” and “older adults.” The search yielded a total of 34 related articles. The selection of BIA equations was based on the reported precision, and it was decided to include those equations that had an *R*^2^ value ≥0.85 and a SEE ≤ 1.8 kg (45). These cut-off points were considered since *R*^2^ is expected to be as close to 1 and SEE as close to zero. An equation with these values can assure considerable precision.

Only BIA equations generated including older adults aged 60 to 90 years, non-institutionalized of any nationality or ethnic group, and with DXA as the reference method were included. Also, the BIA equations had to include any of the following variables: age in completed years, sex, R, Xc, resistance index (height in cm^2^/R), or body weight. Age, sex, and body weight are both clinically and statistically associated with ASM, and together with BIA variables, the predictive model can yield more precise and accurate results of the ASM. Finally, the selected equations must have been generated with a single or multi-frequency bioimpedance model. Also, there was no discrimination regarding the method of generation and validation of the equations, nor the nutritional status of the subjects that integrated the generation or validation sample.

### Statistical analysis

The data was analyzed using STATA version 16 (StataCorp LP, TX, USA). An exploratory analysis of the primary data was carried out to observe the behavior of the data and detect atypical data or outliers. The significance of the differences between men and women was determined using an independent sample *t*-test and the results are presented as mean ± standard deviation. To test if the differences between the ASM measured by DXA Lunar and Hologic were different from zero, a paired *t*-test was used in the sample of 70 adults.

Regarding the validation procedure, the agreement between methods was evaluated using the Bland and Altman procedure, which considers that the average of the two methods is the best estimator. Objectively agreement was tested by a paired *t* test and by simple linear regression analysis. The paired *t*-test assessed if the mean differences between the estimation of each equation and the ASM measurement by DXA were statistically different from zero, and the simple linear regression analysis, which assessed the homogeneity of the dependent variable. To visually analyze the mean of the differences and the distribution of the differences between methods, Bland and Altman (46) plots were incorporated.

Taking the above into account, the following criteria was established to test agreement between methods: The paired *t* test must prove that the mean of the difference between each BIA equation and DXA as reference method is equal to zero (a *p*-value > 0.05 is expected). Additionally, the simple regression analysis must test that the differences are randomly distributed. For this, a *p*-value of the beta coefficient >0.05 was expected. This would prove the homogeneity of the bias, that is, the homogeneous distribution of the differences along the spectrum of the mean of ASM between methods. If these two conditions were met, agreement was accomplished, meaning that the BIA equation can be considered as an interchangeable method to DXA to assess ASM in this large sample of non-Caucasian older adults. This methodology to establish agreement has been described and applied in other validation studies (33, 47).

This bias analysis supports or rejects the possibility of deriving a CF. In order to propose one, the bias distribution must be homogeneous, and the mean of the differences must be different from zero. That is, the *p*-value of β of the simple linear regression must be >0.05, and the *p*-value of the paired *t*-test must be <0.05. If so, the equation can be corrected by subtracting or adding the mean difference to the respective equation. This CF does not change the behavior of the variables included in the equation, but it makes it possible to reduce the average of the differences (bias) in the estimates at group level. This correction has been proposed in other studies (33, 48), and has provided the opportunity to improve the estimates according to the equations where applicable.

## Results

The initial sample made up of all the subjects participating in the previously mentioned studies was of 649 participants. Ninety-five volunteers were excluded due to lack of BIA data. After removing subjects with incomplete or duplicate records, or with outliers (*n* = 7), a sample of 547 subjects who had complete data on the variables required for this external validation study was formed. The sample consists of 338 women (61.8%) and 209 men (38.2%), with a mean age of 70 years (age range: 60–94 years). Some of them reported a previous diagnosis of hypertension, controlled type 2 diabetes, and dyslipidemia, with their respective pharmacological control. Other diseases reported were colitis, gastritis, bronchitis, rheumatoid arthritis, bronchial asthma, or controlled hypothyroidism, with stable weight according to self-report.

The mean value of BMI was 27.9 kg/m^2^ (range: 16–44.4 kg/m^2^). According to their BMI classification, 6 subjects were underweight (1.1%), 129 had a normal weight (23.6%), 259 were overweight (47.4%), and 153 had obesity (27.9%). The mean value of ASM in the whole sample was of 17.4 kg. According to the DXA model, the mean ASM measured by DXA Hologic was 15.9 kg, while that measured by Lunar DXA was 18.2 kg. The general characteristics of both samples are found in Table 1.

**Table 1:** General characteristics of the sample.

**Variables**	**Total sample of subjects measured with both DXA models**		**Sample of subjects measured with DXA Hologic**		**Sample of subjects measured with DXA Lunar**	
	**Men** **(*n* = 209)**	**Women** **(*n* = 338)**	**Men** **(*n* = 40)**	**Women** **(*n* = 152)**	**Men** **(*n* = 169)**	**Women** **(*n* = 186)**
Age (years)	70.1 ± 6.8	69.3 ± 6.7	71.7 ± 7.8	70.3 ± 6.9*	69.6 ± 6.4	68.5 ± 6.5
Weight (kg)	76.1 ± 11.7	69.2 ± 11.6*	80.1 ± 12.9	71.5 ± 12.5*	75.2 ± 11.3	67.3 ± 10.6*
Height (m)	1.6 ± 0.1	1.5 ± 0.1*	1.6 ± 0.1	1.5 ± 0.1*	1.6 ± 0.1	1.5 ± 0.1*
BMI (kg/m^2^)	26.7 ± 3.5	28.6 ± 4.4*	27.7 ± 3.7	29.6 ± 4.6*	26.5 ± 3.4	27.8 ± 4.1*
FMI (kg/m^2^)	8.1 ± 2.6	12.5 ± 3.3*	9.1 ± 2.6	13.1 ± 3.2*	7.8 ± 2.5	11.9 ± 3.2*
WC (cm)	97.8 ± 10.4	98.5 ± 12.1	100.8 ± 11.6	99.4 ± 12.1	97.1 ± 10	97.7 ± 11.9
Resistance (Ω)	505.4 ± 62.7	585.6 ± 73.9*	489.3 ± 51.1	560.7 ± 67.2*	509.2 ± 64.7	605.9 ± 73.2*
Reactance (Ω)	49.4 ± 9.2	50.9 ± 9.8	46.9 ± 10.1	48.2 ± 9.5	50 ± 8.9	53.2 ± 9.4*
RI (cm^2^/R)	57.2 ± 8.2	41.9 ± 6.2*	59.6 ± 8.1	43.5 ± 6.3*	56.6 ± 8.1	40.5 ± 5.8*
ASM _DXA_ (kg)	21.4 ± 3.1	14.9 ± 2.4*	20.8 ± 3.1	14.6 ± 2.6*	21.6 ± 3.1	15.1 ± 2.1*
ASM _Kim_ (kg)	15.2 ± 1.4	10.3 ± 1.2*	15.6 ± 1.6	10.6 ± 1.3*	15.1 ± 1.4	10.1 ± 1.1*
ASM _Kyle_ (kg)	22.2 ± 2.9	15.6 ± 2.4*	23.1 ± 3.1	16.1 ± 2.5*	22.1 ± 2.8	15.2 ± 2.1*
ASM _Rangel_ (kg)	21.2 ± 2.5	14.4 ± 2.1*	22.1 ± 2.5	15.1 ± 2.1*	21.1 ± 2.4	14.1 ± 1.9*
ASM _Sergi_ (kg)	20.8 ± 2.6	15.3 ± 2.1*	21.5 ± 2.7	15.8 ± 2.3*	20.6 ± 2.5	15.1 ± 1.9*
ASM _Toselli_ (kg)	22.2 ± 2.1	15.1 ± 1.8*	22.8 ± 2.3	15.5 ± 1.9*	21.6 ± 2.1	14.4 ± 1.6*
ASM_Yoshida_ (kg)	24.8 ± 3.3	18.2 ± 2.5*	26.1 ± 3.5	18.8 ± 2.6*	24.5 ± 3.2	17.7 ± 2.2*

^*^p < 0.05 when performing the t-test for independent samples between sex. Means ± standard deviation. BMI, body mass index; FMI, fat mass index; WC, waist circumference; RI, resistance index; ASM, appendicular skeletal muscle mass.

### Selected BIA predictive models

Regarding the BIA equations to estimate the ASM, a total of 25 equations were found, of which 10 were generated in older adults. Of these, only 5 had reported an internal validation process, and 6 have been externally validated in other studies. Only 6 equations which met the selection criteria were selected: Kim's, Kyle's, Rangel-Peniche's, Sergi's, Toselli's and Yoshida's equations. The characteristics of these equations are shown in Table 2. These equations were applied to the complete sample, and with this, the variables ASM_Kim_, ASM_Kyle_, ASM_Rangel_, ASM_Sergi_, ASM_Toselli_ and ASM_Yoshida_ were obtained. Importantly, Kim's and Toselli's equations generated with DXA Lunar, were tested on subjects measured with DXA Lunar, while BIA equations generated using DXA Hologic as the reference method, were tested on those measured with that model. This, in order to eliminate the effect or possible bias due to DXA model in this validation procedure.

**Table 2:** Selected equations and their characteristics.

**Reference**	**Equations**	**n/sex**	**Age (years)**	**BMI (kg/m^2^)**	**ASM DXA (kg)**	***R^2^***	**SEE (kg)**	**DXA model used**
Kim et al. (8)	*ASM*_(*kg*)_ = (0.104 × *RI*) + (0.050 × *age*) + (2.954 × *sex*) + (0.055 × *weight*)+5.663	483/M 642/W	73.5 ± 5.6	24.4 ± 3.2	M: 20.1 ± 2.6 W: 13.6 ± 1.8	0.88	1.35	DXA Lunar Corporation, Madison, WI
Toselli et al. (9)	*ASM*_(*kg*)_ = 5.982+(0.188 × *RI*) + (0.014 × *WC*) + (0.046 × *weight*) + (3.881 × *sex*)−(0.053 × *age*)	26/M 92/W	71.2 ± 7.2	27.9 ± 5.1	16.2 ± 3.5	0.86	1.35	Lunar DPX-MD
Kyle et al. (10)	*ASM*_(*kg*)_ = −4.211+(0.267 × *RI*) + (0.095 × *weight*) + (1.909 × *sex*) + (−0.012 × *age*) + (0.058 × *Xc*)	459/M 311/W	20-94	V: 25 ± 3.2 P: 24.6 ± 4.4	M-V: 25.8 ± 3.6 M-P: 22.1 ± 2.8 W-V: 17.3 ± 2.5 W-P: 15.2 ± 2.8	V: 0.95 P: 0.91	V: 1.12 P: 1.5	DXA Hologic QDR4500A
Rangel-Peniche et al. (11)	*ASM*_(*kg*)_ = −0.05376+(0.2394 × *RI*) + (2.708 × *sex*) + (0.065 × *weight*)	55/M 158/W	68 ± 5.9	-	15 ± 3.4	0.91	1.01	DXA Hologic Explorer QDR-4500W
Sergi et al. (12)	*ASM*_(*kg*)_ = 3.964+(0.227 × *RI*) + (0.095 × *weight*) + (1.384 × *sex*) + (0.064 × *Xc*)	117/M 179/W	71.4 ± 5.4	27.0 ± 3.4	18.6 ± 4.1	0.92	1.14	DXA Hologic QDR Discovery A
Yoshida et al. (13)	*MME*_*men* (*kg*)_ = (0.197 × *RI*) + (0.179 × *weight*)−0.019 *MME*_*women* (*kg*)_ = (0.211 × *RI*) + (0.170 × *weight*)+ 0.881	141/M, 109/W.	73.5 ± 5.6	23.4 ± 3.4	17.8 ± 3.8	M:0.87 W:0.89	M: 0.98 W: 0.81	DXA Hologic QDR 4500 A

Means ± standard deviation. BMI, body mass index; R^2^, coefficient of determination; SEE, standard error of the estimate; ASM, total appendicular skeletal muscle; RI, resistance index; Xc, reactance; WC, waist circumference; M, men; W, women; V, volunteers; P, patients; Sex variable: 0 for female, 1 for male. Weight in kilograms. ASM, appendicular skeletal muscle mass; n, number of subjects.

### Differences in ASM measurements between DXA lunar and DXA hologic

The results of the paired *t*-test between the measurements by both DXA models in the subsample of 70 subjects, showed a mean difference different from zero (16.7 kg using DXA Hologic vs. 17.1 kg using DXA Lunar, mean difference of−0.4 kg; *p* < 0.001). These differences between DXA models support the decision to validate the equations according to the DXA model taken as reference, since the measurements between both models are not interchangeable.

### Validation of the BIA equations to estimate ASM

**Figure 1:**
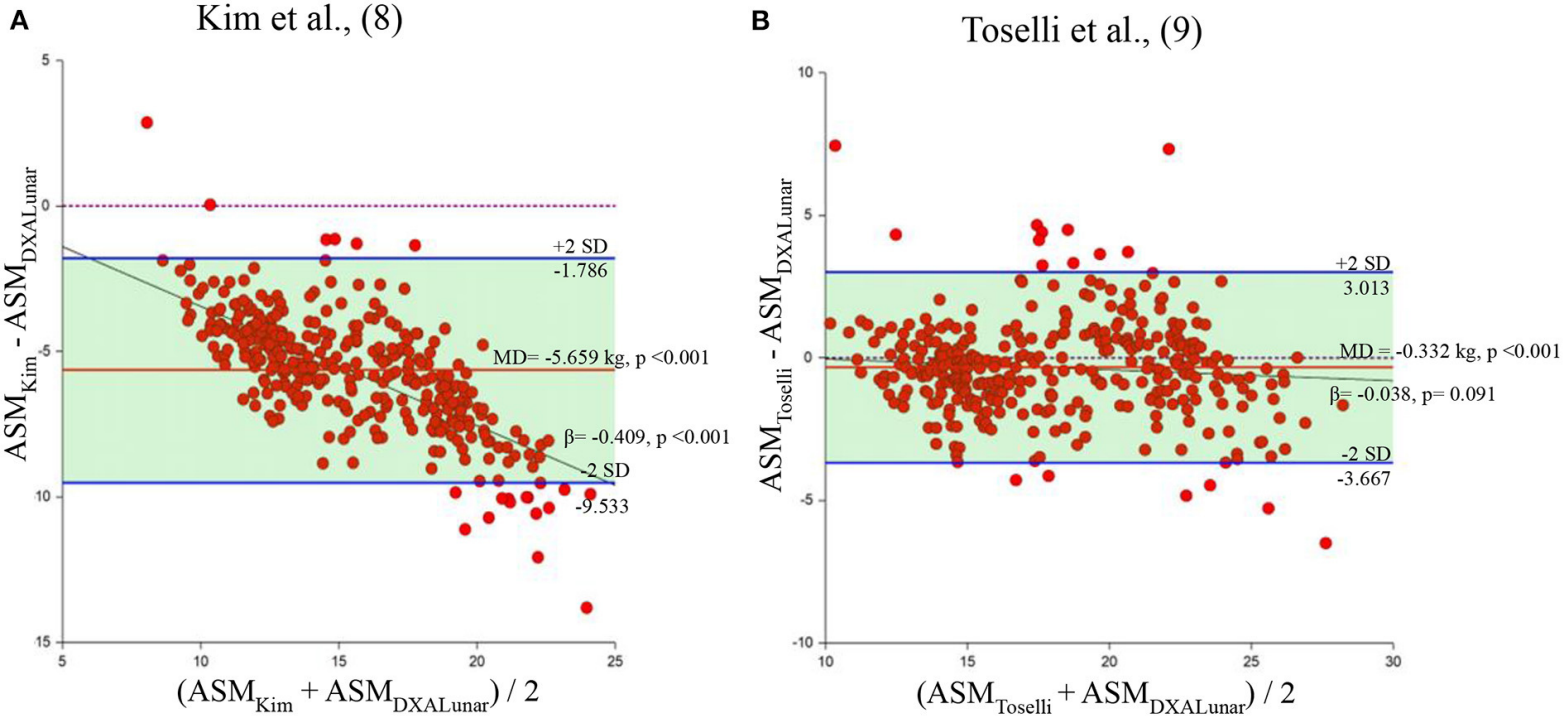
Bland and Altman plots of the equations generated using DXA Lunar. Behavior of the mean difference against the mean of the measurements between the equations of Kim et al., (8) and Toselli et al., (9) and DXA Lunar. Solid red lines indicate the mean difference. Solid blue lines indicate limits of agreement. Solid black lines indicate the regression line. Dotted line indicates zero. ASM, appendicular skeletal muscle mass; MD, mean of the differences. **(A)** Kim et al. (8). **(B)** Toselli et al. (9).

**Figure 2:**
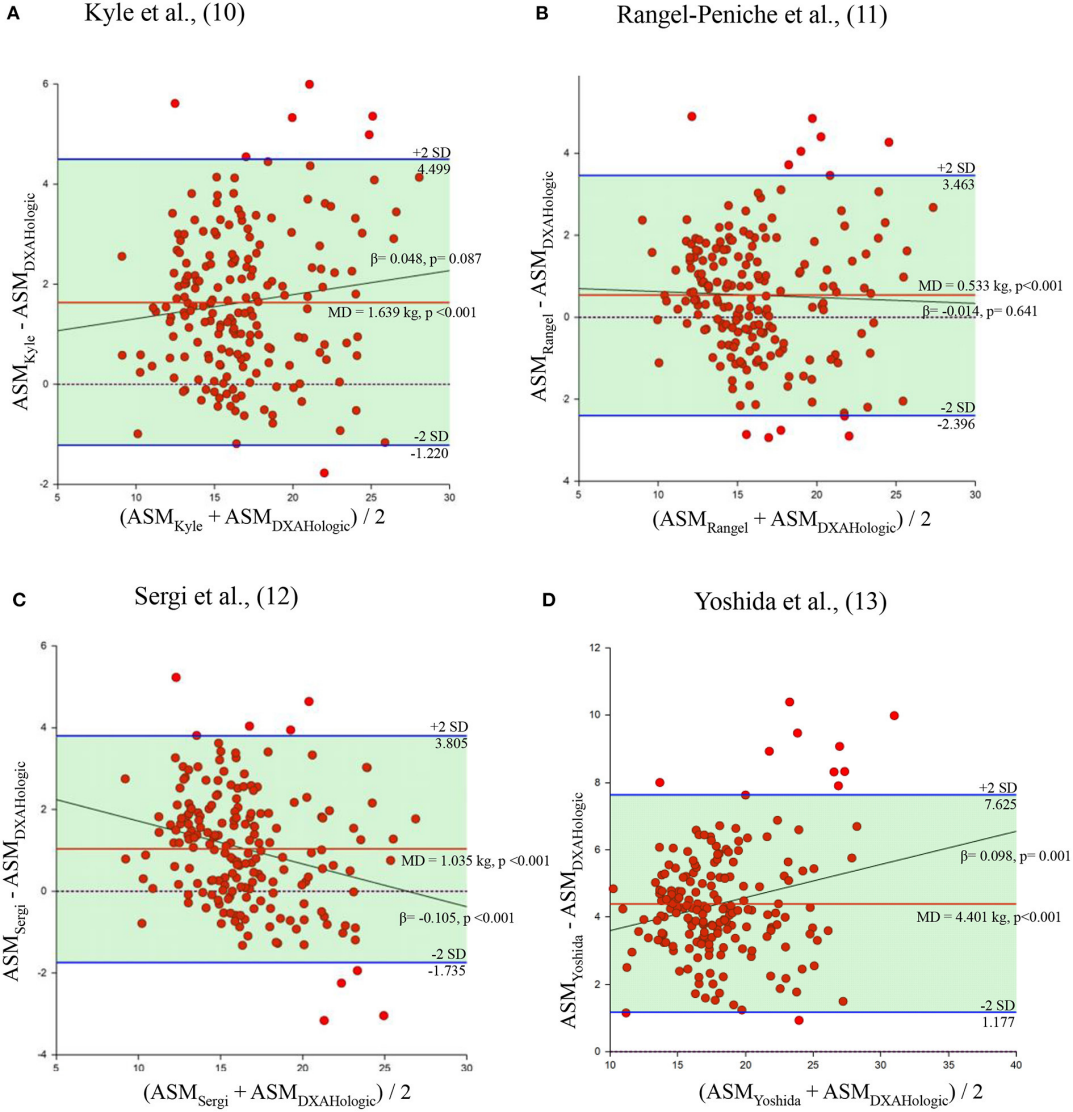
Bland and Altman plots of the equations generated using DXA Hologic. Behavior of the mean difference against the mean of the measurements between the equations of Kyle et al., (10), Rangel-Peniche et al., (11), Sergi et al., (12) and Yoshida et al., (13), and DXA Hologic. Solid red lines indicate the mean difference. Solid blue lines indicate limits of agreement. Solid black lines indicate the regression line. Dotted line indicates zero. ASM, appendicular skeletal muscle mass; MD, mean of the differences. **(A)** Kyle et al. (10). **(B)** Rangel-Peniche et al. (11). **(C)** Sergi et al. (12). **(D)** Yoshida et al. (13).

The mean value of ASM estimated by the Kim's and Toselli's equations in the sample of subjects measured by DXA Lunar was 12.5 and 17.8 kg, respectively. Regarding the Kyle, Rangel-Peniche, Sergi and Yoshida equations, the mean value of ASM was 17.6, 16.5, 17.0 and 20.4 kg, respectively. When each of the six BIA equations were compared with their respective reference method, the mean of the differences between the Kim, Toselli, Kyle, Rangel, Sergi and Yoshida equations and the ASM_DXA_ was−5.6,−0.3, 1.6, 0.5, 1.0 and 4.4 kg, respectively (Figures 1, 2). Clearly, these results indicate that 2 equations underestimated ASM_DXA_, while 4 overestimated it (Table 3).

**Table 3:** Validation data of the six BIA equations.

**Equations**	**Estimated ASM (kg)**	**Mean difference (kg)**	**Limits of agreement**	**95% Confidence interval**	**β of SLR**	***p-*value of SLR**
Kim	12.5	−5.6	−9.5,−1.7	−5.8,−5.4	−0.409	<0.001
Toselli	17.8	−0.3	−3.6, 3.0	−0.5,−0.1	−0.038	0.091
Kyle	17.6	1.6	−1.2, 4.4	1.4, 1.8	0.048	0.087
Rangel-Peniche	16.5	0.5	−2.3, 3.4	0.3, 0.7	−0.014	0.641
Sergi	17.0	1.0	−1.7, 3.8	0.8, 1.2	−0.105	<0.001
Yoshida	20.3	4.4	1.1, 7.6	4.1, 4.6	0.098	0.001

SLR, simple linear regression between the mean difference and the mean of ASM by both methods.

The statistical analysis showed that the mean of the differences between each of the equations and the ASM_DXA_ was statistically different from zero (*p* < 0.001) (Table 4). However, Toselli's, Kyle's and Rangel-Peniche's equations showed a homogeneous distribution of the bias over the entire range of ASM values between methods (β = −0.038, *p* = 0.091; β = 0.048, *p* = 0.087; and β = −0.014; *p* = 0.641, respectively) (Table 3; Figure 1). This indicates that these equations do not significantly underestimate or overestimate as ASM increases. Having a homogeneous bias allows us to suggest a correction factor, which could correct the significant differences found in the paired *t* tests in these three equations.

**Table 4:** Comparison of the mean values of the estimated ASM and the ASM_DXA_.

**DXA model**	**Equations**	**Estimated ASM (kg)**	**ASM DXA (kg)**	***p*-value**
Lunar	Kim et al. (8)	12.5	18.2	<0.001
	Toselli et al. (9)	17.8		<0.001
Hologic	Kyle et al. (10)	17.6	15.9	<0.001
	Rangel- Peniche et al. (11)	16.5		<0.001
	Sergi et al. (12)	17.0		<0.001
	Yoshida et al. (13)	20.3		<0.001

This wasn't possible for Kim's, Sergi's and Yoshida's equations. In addition to the paired *t*-tests results, the simple linear regression showed a non-homogeneous bias in these equations (β = 0.409, *p* < 0.001; β = −0.105, *p* < 0.001; and β = 0.098, *p* = 0.001, respectively) (Tables 3, 4). In these cases, the overestimation or underestimation of these equations as the ASM increases is significant, so they cannot be corrected.

### Derivation of the correction factors and validation of the corrected equations

Considering the finding of homogeneous bias, correction factors were proposed by considering the mean difference between DXA and both equations. The bias of each one of the equations was subtracted or added as following:


$$\begin{array}{l} ASM_{ToselliCF}\;=5.982\;+\;\left(0.188\;\times \;RI\right)\;+\;(0.014\\ \;\times \;WC)\;+\;\left(0.046\;\times \;weight\right)\;+\;\left(3.881\times sex\right)\\ \;-\;\left(0.053\;\times \;age\right)\;+\;0.332\\ ASM_{KyleCF}\;=\;-4.211\;+\;\left(0.267\times RI\right)\;+\;\left(0.095\times weight\right)\\ \;+\;\left(1.909\;\times \;sex\right)\;+\;\left(-0.012\;\times \;age\right)\;+\;(0.058\\ \;\times \;reactance)\;-\;1.639\\ ASM_{RangelCF}\;=\;-0.05376\;+\;\left(0.2394\;\times \;RI\right)\;+\;\left(2.708\times sex\right)\\ \;+\left(0.065\;\times \;weight\right)\;-\;0.533 \end{array}$$

ASM_ToselliCF_, corrected Toselli's equation. ASM_KyleCF_, corrected Kyle's equation. ASM_RangelCF_, corrected Rangel-Peniche's equation. RI, resistance index (height in cm^2^/resistance). WC, waist circumference in cm. Weight in kilograms. Sex: 0 for women and 1 for men. Age in years.

The mean value of ASM estimated by the corrected Toselli's equation (Toselli_CF_) in the sample of subjects measured by DXA Lunar was 18.2 kg. On the other hand, the mean value of ASM estimated by the corrected Kyle's equation (Kyle_CF_) and the corrected Rangel-Peniche's equation (Rangel_CF_) in the sample of subjects measured by DXA Hologic was 15.9 kg for both equations. When these three corrected BIA equations were compared with their respective reference method, the mean differences were less than 0.01 kg.

**Figure 3:**
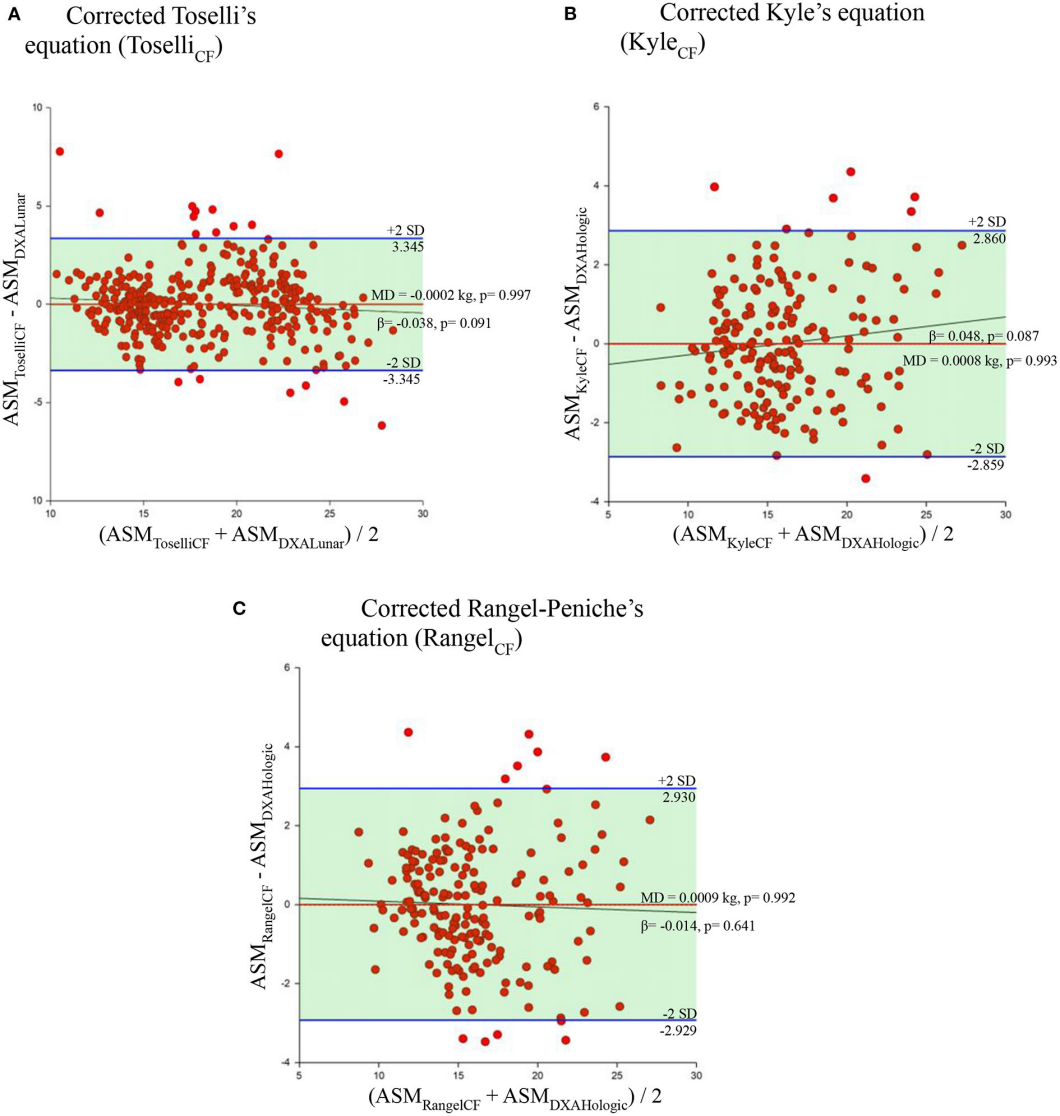
Bland and Altman plots and simple linear regression of the selected equations applying the correction factors. Behavior of the mean difference against the mean of the measurements between the corrected equations and their respective reference method. Solid red line indicates the mean difference. Solid blue line indicates the limits of agreement. Solid black line indicates the regression line. Dotted line indicates zero. ASM, appendicular skeletal muscle mass. MD, mean of the differences. **(A)** Corrected Toselli's equation (Toselli_CF_). **(B)** Corrected Kyle's equation (Kyle_CF_). **(C)** Corrected Rangel-Peniche's equation (Rangel_CF_).

By carrying out the same tests applied previously (paired *t* test and simple linear regression), and considering the criteria to determine agreement, it was possible to achieve agreement between the three corrected BIA equations and the ASM_DXA_. The paired *t*-tests (Table 5) showed that the mean differences between these BIA equations and the ASM_DXA_ were not statistically different from zero (*p* = 0.997 for Toselli's corrected equation, *p* = 0.993 for Kim's corrected equation and 0.992 for Rangel-Peniche's corrected equation). The homogeneous bias distribution of these BIA estimations remained the same graphically and objectively, tested by simple linear regression (β = −0.038, *p* = 0.091; β = 0.048, *p* = 0.087; and β = −0.014, *p* = 0.641, respectively) and in the Bland and Altman plot, it was possible to observe a mean difference almost above the zero line in these three corrected equations (Figure 3, Table 6). This analysis gave us three corrected equations with a bias very close to zero, which is not statistically significant, and which maintained a homogeneous bias in the estimation.

**Table 5:** Comparison of the mean values of the estimated ASM and the ASM_DXA_.

**DXA** **model**	**Equations**	**Estimated ASM (kg)**	**ASM DXA (kg)**	***p*-value**
Lunar	Toselli_CF_	18.2	18.2	0.997
Hologic	Kyle_CF_	15.9	15.9	0.993
	Rangel-Peniche_CF_	15.9		0.992

Toselli_CF_, corrected Toselli's equation; Kyle_CF_, Kyle's corrected equation; Rangel-Peniche_CF_, corrected Rangel-Peniche's equation; Eq, equation.

**Table 6:** Validation data of the three corrected BIA equations.

**Corrected equation**	**Estimated ASM (kg)**	**Mean difference (kg)**	**Limits of agreement**	**95% Confidence interval**	**β of SLR**	***p*-value of SLR**
Toselli_CF_	18.2	−0.0002	−3.3, 3.3	−0.1, 0.1	−0.038	0.091
Kyle_CF_	15.9	0.0008	−2.8, 2.8	−0.2, 0.2	0.048	0.087
Rangel-Peniche_CF_	15.9	0.0009	−2.9, 2.9	−0.2, 0.2	−0.014	0.641

SLR, simple linear regression between the mean difference and the mean of ASM by both methods; Toselli_CF_, Toselli's corrected equation; Kyle_CF_, Kyle's corrected equation; Rangel_CF_, Rangel-Peniche's corrected equation.

## Discussion

The purpose of this study was to validate some published BIA equations for estimating ASM. None of these BIA equations met the criteria for agreement in this sample. However, the analysis of bias permitted to derive CFs, which, when applied to some equations, showed agreement with DXA. A valid corrected equation for this group of older adults can be a useful tool for epidemiological studies. To the best of our knowledge, in Mexico, low muscle mass has only been assessed at the national level using calf circumference (49). From our perspective, estimating it with accurate and practical tools, such as BIA equations could guarantee a better estimate of skeletal muscle, particularly ASM.

All the BIA equations selected for this study have already been tested in other populations previously, where they were discarded for its inaccuracy in certain populations due to the difference in age ranges (11, 12, 21), nutritional status (20, 50), differences in body composition and anthropometry measurements related to ethnicity (18), health status (19), differences in functional status (14), or BIA device employed (18).

For example, in other external validation studies (18, 20), Kim's equation was found to have the highest mean difference compared to DXA Lunar ASM estimations. In these studies, authors discuss that it is most likely due to the fact that it was developed for an Asian population, but also because the authors used a multifrequency bioimpedance device, operating at a single frequency of 250 Hz. It is already well recognized, that low frequencies predominantly measure extracellular water. At higher frequencies, in contrast, cell membranes are permeable to current, so both intracellular and extracellular water are measured (51). In this way, it is understood that multifrequency devices measure body composition in a slightly different way. In our study, the Kim equation yielded the highest mean difference of all (-5.659 kg), followed by the Yoshida equation (4.401 kg). Both equations were generated in older Asian adults and using multi-frequency BIA devices, thus, we hypothesize that these two characteristics may have been an important factor contributing to bias in this sample as well.

Sergi's equation was generated in Caucasian subjects, and it only included older adults for its generation process. Even though their generation sample has very similar characteristics to ours, the equation had a very high bias, and like the others, the mean of the differences was significant. It is important to remember that several studies have described the differences in body composition between different ethnic groups (42, 52, 53), which could also have contributed to the bias of this equation as well. In addition to the high and significant bias found in these aforementioned equations, the Kim, Yoshida, and Sergi equations did not have a homogeneous bias distribution (β coefficient *p*-value > 0.05). This did not allow a correction factor to be proposed for these models.

Kyle's equation was developed for Swiss adults in the age range of 22 to 94 years. Many studies have tried to validate it in external validation protocols. In almost all validation studies (11, 12, 14, 16, 18, 19, 21–23, 50, 54), the equation has overestimated the ASM in different conditions, which the authors consider is due to the fact that it is not specific for a particular age group. Therefore, this equation is usually discarded for use in certain populations. In our study, this equation overestimated 1.639 kg, and we agree that this was probably because it was not generated including subjects similar to those in this sample, and that it was not specific for older adults.

Toselli and Rangel-Peniche equations were the ones with the mean of the differences closest to zero (-0.332 and 0.533 kg, respectively). In the case of the Rangel-Peniche equation, this must be since it was developed in a group of individuals of the same nationality as our sample. Despite this, this equation does not meet the established criteria for agreement in this sample of older adults from the northwest of the same country. This confirms the nature of the equations to be specific for the population where it was generated and very similar populations. In fact, another study by Rangel-Peniche et al. (29) evaluated the differences in body composition of older adults from central Mexico and older adults from the northwest of the country. After adjusting for age, body weight, height, health status, estimated energy expenditure, and some demographic variables, ASM and the appendicular muscle mass index in older adults from central Mexico were significantly higher compared to the older adults from the northwest of Mexico. This could be one reason why Rangel-Peniche's equation was not valid for our sample. In other studies, such as the one by Yu et al. (18), the Rangel-Peniche's equation also underestimated the ASM when applied to Australian adults, with a mean error of 1.82 kg. In another study (19), it overestimated approximately 0.51 kg when applied to subjects with anorexia. In the study by Coëffier et al. (20) the equation had a mean difference even futher from zero, of−2.68 kg. Due to these values, these studies have decided to rule out the use of this equation.

On the other hand, this is the first study to externally validate Toselli's equation. This model, which includes waist circumference among the predictor variables, turned out to have a very low bias in our sample (-0.332 kg). In their study, the authors discuss the relationship between waist circumference and ASM. We believe that having taken this variable into account in this model and applying it to a sample with a high mean waist circumference, could be the reason why it had the smallest mean difference.

According to our results, none of the selected equations was valid for older adults from the northwest of Mexico. However, an important finding achieved when analyzing the bias of the equations, is that we realized that the Toselli, Rangel-Peniche and Kyle equations had a homogeneous bias. This allowed them to be further improved to yield accurate data in this sample of older Mexican adults. By deriving a correction factor for Toselli's, Kyle's and Rangel-Peniche's equations, precise, accurate, and bias-free ASM estimates were obtained. Importantly, this was possible after the analysis of the bias in this external validation study. This turned out to be a very useful strategy to use the existing equations in the literature, and thus not contribute to the development of more equations, which would have been generated unjustifiably and that, as mentioned in the systematic review by Beaudart et al. (25) would have been redundant.

This study has several advantages: to our knowledge, it is the first study to propose correction factors for BIA equations to estimate ASM, derived from a validation study with a large sample that included subjects of a wide nutritional range, age range, physically independent and without uncontrolled diseases that affected body composition. Likewise, it is the first study that considers the DXA model in the validation process. Many external validation studies have treated the DXA model indistinctly, despite the differences that are already recognized in the literature (31, 32, 55–57). In this study, in addition to considering these differences, we tested if the measurements taken by both DXA models were different in a subsample of subjects. Once confirmed, we chose to separate the validation according to the DXA model: the equations generated with a model, were applied only in subjects measured with that same model. This reduces the influence of the DXA model in the validation process, which could have been an important contributing bias factor.

Another advantage is that this validation confirms that single frequency bioimpedance devices are a valid tool for ASM estimation compared to DXA. These models are cheaper and more practical compared to others, and they can be a portable alternative for epidemiological studies.

A final advantage that we find are the criteria established in this article to determine agreement between methods. When assessing other validation studies, we noticed that some of them only carry out paired *t*-tests between methods, some use the pure error, or the Pearson or Lin coefficient. Some others are satisfied with only determining which was the lowest mean error of the selected equations. We also notice that most studies do not analyze the bias distribution. We opted for the criteria mentioned in the Materials and Methods section, because, by adding paired *t*-tests and simple linear regression to the statistical methods, we address more than what is included in the Bland and Altman plot, testing agreement not only subjectively, but also objectively. These steps should be fundamental in validating equations.

One disadvantage of this study is that, due to its nature, the CF may not be generalizable to other populations. Likewise, this CF could be more viable for overweight and obese subjects, since approximately 75.2% of our sample in this validation study is made up of these subjects. A very little percentage of our subjects is made up of low-weight subjects, so it could be less valid for this group of individuals. Another disadvantage is that, despite that this study has a larger sample compared to others published, our sample is not representative or randomized, so our results are only valid in this sample, and we hypothesize that it may be valid in subjects with similar characteristics.

Moreover, it is important to mention that in this study, agreement was proven statistically, and this is not synonymous with clinical significance. It is notorious that, when applying these CFs, there are no changes or improvements in the amplitude of the limits of agreement of the estimations, and it only allows the reduction of the mean difference. Given this, the corrected equations by this CFs are only useful for estimating mean values of ASM on populations and are not valid if applied at the individual level, since the estimates exceed clinically significant physiological values. Because of this, first, we recommend exploring through regression models, which are the variables associated with bias in each of the equations, to obtain a broader picture of the main contributing bias factors. Subsequently, knowing the variables associated with the bias in these equations, we recommend generating more complex correction equations, to obtain values closer to the real ones at the individual level.

We also recommend validating these CFs on an independent sample, as long as the DXA model used as the reference method is considered. Furthermore, we consider that clinically acceptable limits of accuracy need to be defined when estimating the ASM.

## Conclusion

None of the published BIA equations met the criteria to achieve agreement with DXA. However, the bias analysis done after stratifying by DXA model, was determinant to derive and apply correction factors to Toselli's (generated with DXA Lunar), Kyle's and Rangel-Peniche's equations (generated with DXA Hologic). Incorporating the correction factors to the corresponding BIA equations showed an extremely low bias. Therefore, these three corrected BIA equations could be used to estimate the mean values of ASM at group level in older adults from the northwest of Mexico.

## Data availability statement

The data analyzed in this study is subject to the following licenses/restrictions: The database from which this article was derived, is available only by request. Requests to access these datasets should be directed to HA-M, helio@ciad.mx.

## Ethics statement

Ethical review and approval was not required for the study on human participants in accordance with the local legislation and institutional requirements. The patients/participants provided their written informed consent to participate in this study.

## Author contributions

HA-M: design. MC-R and HA-M: drafting of manuscript. MC-R, HA-M, JE-R, and RG-A: data analysis. HA-M, RG-A, MR-T, RU-R, DR-P, and GF-P: recruitment and data collection. All authors contributed to the article and approved the submitted version.

## Funding

The databases employed in this study were collected from five projects funded by the National Science and Technology Council, Mexico (CONACYT; J37891-M, S0008-2010-1-140157 and CB-2013-01/000000000221664) and IAEA (Research contract No. 12694/R0 and Research contract No. RLA/6/07).

## Conflict of interest

The authors declare that the research was conducted in the absence of any commercial or financial relationships that could be construed as a potential conflict of interest.

## Publisher's note

All claims expressed in this article are solely those of the authors and do not necessarily represent those of their affiliated organizations, or those of the publisher, the editors and the reviewers. Any product that may be evaluated in this article, or claim that may be made by its manufacturer, is not guaranteed or endorsed by the publisher.
